# P213: The evaluation of infection control activities and analysis of influence factors in healthcare setting in Korea

**DOI:** 10.1186/2047-2994-2-S1-P213

**Published:** 2013-06-20

**Authors:** H Koo

**Affiliations:** 1 Infectious disease control, Korea CDC, Cheongwon-gun, Korea, Republic Of

## Introduction

The healthcare associated infection (HAI) is one of the most serious threat in patient safety in the process of medical services. The greater part of these unexpected infections could be prevented with effective infection control activities.

## Objectives

The aim of this study was to evaluate infection control activities and analyze the influence factors in healthcare setting in Korea.

## Methods

The study was conducted in general acute care hospitals in Korea. In 2012, we surveyed the infection control activities in hospital which operate the infection control organization by the law. To measure the infection control activities of hospitals, we applied the the Infection Control Assessment Tool (ICAT) 2009 restrictively to the surveyed data.

We calculated the infection control activity scores in the indexes of infection control infrastructure, surveillance, compliance of guidelines, education, antibiotics use and indicator monitoring activities. Finally we analyze the influence factors to the total activity scores.

## Results

Among 154 hospitals operating infection control organization, 127 hospitals (82.0%) were participated. The mean score was 77.5 in the range of 30 to 95 (total possible score was 100). The variables affect to the total score are university hospital, nurse staffing, work experiences of infection control nurse (ICN), the certification of ICN, antibiotic stewardship and education completion of doctors. In multiple linear regression analysis, the influence factors are antibiotic stewardship (β=8.2, p=0.001), education completion of doctors (β=4.5, p=0.001) and the certification of ICN (β=3.6, p=0.011).

## Conclusion

Our result shows the important factors to measure or evaluate the infection control activity are the antibiotics stewardship, education completion of doctors and the certification of ICN.

## Disclosure of interest

None declared

